# HMG-CoA Reductase Inhibitor Statins Activate the Transcriptional Activity of p53 by Regulating the Expression of TAZ

**DOI:** 10.3390/ph15081015

**Published:** 2022-08-17

**Authors:** Chiharu Miyajima, Yurika Hayakawa, Yasumichi Inoue, Mai Nagasaka, Hidetoshi Hayashi

**Affiliations:** 1 Department of Cell Signaling, Graduate School of Pharmaceutical Sciences, Nagoya City University, Nagoya 467-8603, Japan; 2 Department of Innovative Therapeutics Sciences, Cooperative Major in Nanopharmaceutical Sciences, Graduate School of Pharmaceutical Sciences, Nagoya City University, Nagoya 467-8603, Japan

**Keywords:** simvastatin, TAZ, p53, repositioning

## Abstract

Transcriptional coactivator with PDZ-binding motif (TAZ) is a downstream transcriptional regulator of the Hippo pathway that controls cell growth and differentiation. The aberrant activation of TAZ correlates with a poor prognosis in human cancers, such as breast and colon cancers. We previously demonstrated that TAZ inhibited the tumor suppressor functions of p53 and enhanced cell proliferation. Statins, which are used to treat dyslipidemia, have been reported to suppress the activity of TAZ and exert anti-tumor effects. In the present study, we focused on the regulation of p53 functions by TAZ and investigated whether statins modulate these functions via TAZ. The results obtained suggest that statins, such as simvastatin and fluvastatin, activated the transcriptional function of p53 by suppressing TAZ protein expression. Furthermore, co-treatment with simvastatin and anti-tumor agents that cooperatively activate p53 suppressed cancer cell survival. These results indicate a useful mechanism by which statins enhance the effects of anti-tumor agents through the activation of p53 and may represent a novel approach to cancer therapy.

## 1. Introduction

Transcriptional co-activator with PDZ-binding motif (TAZ) is a transcriptional co-factor downstream of the Hippo pathway that regulates cell proliferation, differentiation, and tissue growth [1,2]. TAZ interacts with transcription factors, such as transcriptional enhancer factor domain (TEAD) in the nucleus, to induce the expression of target genes, including connective tissue growth factor and the anti-apoptosis factor survivin, thereby positively regulating cell proliferation [3]. The aberrant activation of TAZ leads to carcinogenesis due to abnormal cell proliferation. The activation of TAZ has been observed in various human cancers and plays an important role in tumor development, progression, and metastasis [4]. In human clinical cancers, such as breast and colorectal cancers, disruption of the Hippo pathway and TAZ overexpression correlate with a poor patient prognosis [5,6,7].

The tumor suppressor p53 regulates cell proliferation in response to various stresses, including genotoxic stress. The transcription factor p53 is normally bound to the ubiquitin ligase Mdm2, which is expressed at low levels. p53 dissociates from Mdm2 in response to intracellular and extracellular stresses, such as DNA damage. Stabilized and activated p53 promotes the expression of target genes, including the cyclin-dependent kinase inhibitor p21 and the apoptosis-inducing factor p53 up-regulated modulator of apoptosis, and negatively regulates tumor proliferation [8,9]. In clinical cancers, the frequency of abnormalities in the p53 pathway is high, and the loss of p53 functions is closely related to the development and progression of cancer [10,11,12]. Approximately half of all malignancies contain p53 mutations and, in many other tumors, the function of the retained wild-type (WT) p53 protein is compromised. Previous studies reported that the effects of p53 were lost due to binding to molecules that suppress p53 function [13,14]. We identified TAZ as a molecule that negatively regulates p53 functions. TAZ inhibits the binding of p53 to DNA and suppresses its transcriptional activation ability [15].

Statins, which are used to treat hypercholesterolemia, are 3-hydroxy-3-methylglutaryl coenzyme A (HMG-CoA) reductase inhibitors that inhibit the mevalonic acid pathway, thereby reducing cholesterol synthesis [16]. Statins are divided into standard and strong types based on the degree of their cholesterol-lowering effects. In general, standard statins are expected to lower cholesterol by about 15% and strong statins by about 30%. In addition, statins are classified as hydrophilic or lipophilic based on their ability to dissolve in a medium. Hydrophilic statins act more selectively on the liver. Lipophilic statins, on the other hand, can easily permeate cells and thus affect organs throughout the body. Therefore, lipophilic statins have been reported to have effects other than lowering cholesterol, such as promoting angiogenesis via nitric oxide [17], bone formation [18], and anti-inflammatory effects [19]. Simvastatin and fluvastatin are lipophilic standard statins and are the most commonly used statins in clinical practice.

Statins have recently attracted attention for their anti-tumor effects because they induce apoptosis and inhibit the growth of cancer cells [20,21]. Geranylgeranyl diphosphate (GGPP), which is generated by branching from farne-syl-2-phosphate in the mevalonic acid pathway, activates Ras and Rho, which contribute to cell proliferation and differentiation. Rho and Ras subsequently inhibit LATS1/2 functions, involved in TAZ inactivation [22]. Statins induce the phosphorylation and inactivation of TAZ by reducing the production of GGPP, thereby inhibiting cell proliferation [22]. Clinical studies have reported significantly higher survival rates in lung and breast cancer patients taking simvastatin than in those not taking the drug [23,24]. However, the mechanisms by which statins exert their antitumor effects via TAZ remain unclear.

In the present study, we found that the suppression of TAZ functions by simvastatin and fluvastatin induced the activation of p53. Furthermore, the co-treatment of simvastatin with nutlin-3, which inhibits Mdm2 and activates WT p53, efficiently reduced cancer cell viability. These results provide novel insights into the anti-tumor mechanisms of statins and suggest innovative cancer therapies that enhance the therapeutic efficacy of anti-tumor agents.

## 2. Results

### 2.1. Simvastatin and Fluvastatin Suppress TAZ Protein Expression

**Figure 1:**
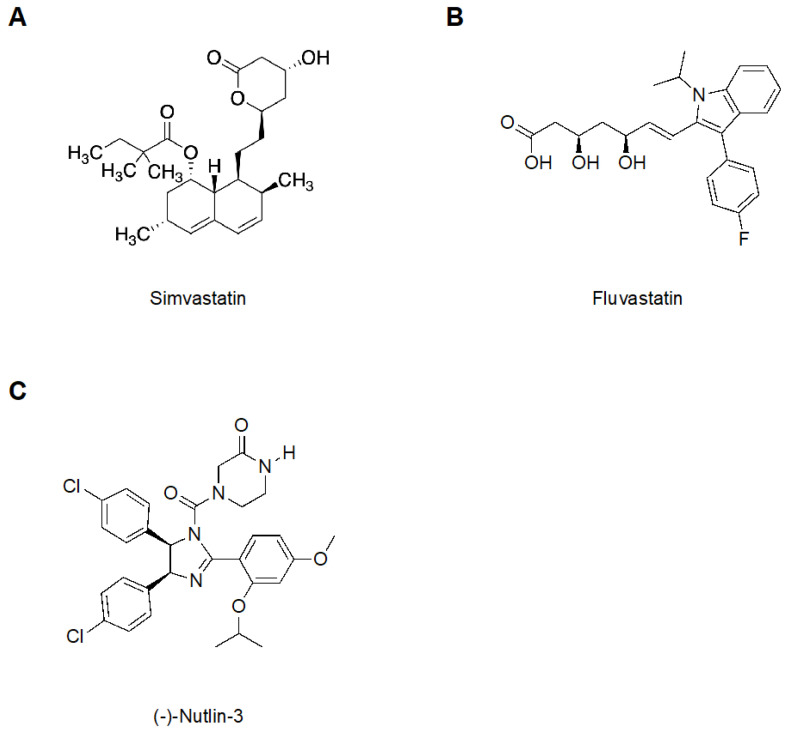
Chemical structures of simvastatin (**A**), fluvastatin (**B**), and (-)-nutlin-3 (**C**).

**Figure 2:**
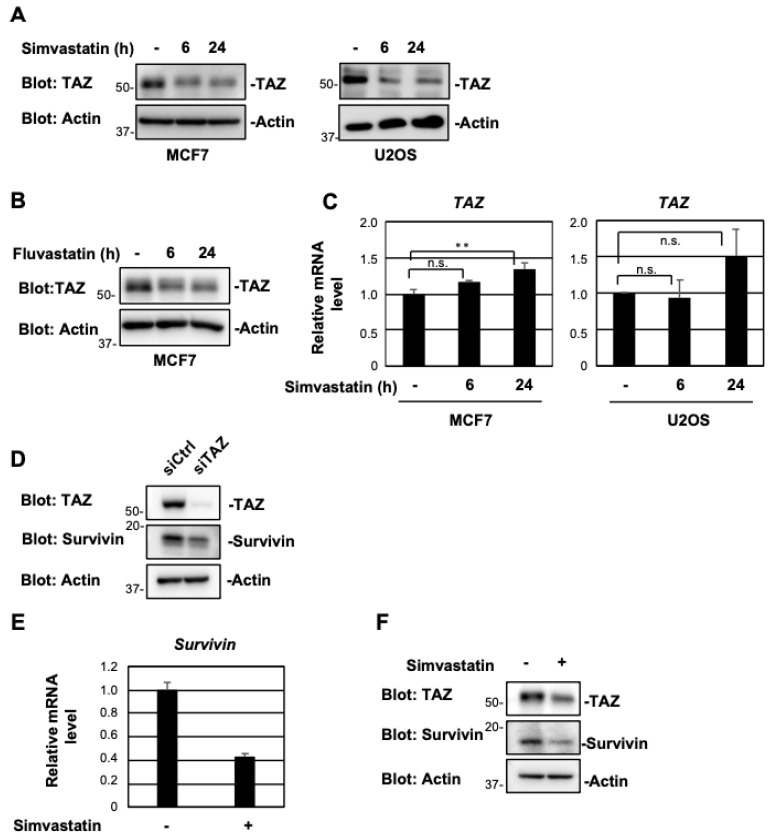
Simvastatin and fluvastatin suppress TAZ protein expression. (**A**) MCF7 and U2OS cells were treated with 10 μM simvastatin for the indicated times. Cell lysates were immunoblotted with the indicated antibodies. (**B**) MCF7 cells were treated with 10 μM fluvastatin for the indicated times. Cell lysates were then analyzed as in (**A**). (**C**) Cells were treated with 10 μM simvastatin and the expression of each gene was assessed by RT-qPCR. The expression levels of *TAZ* were normalized against the expression level of *β-actin* mRNA. Results are shown as the mean ± S.D. (*n* = 3). (**D**) MCF7 cells were transfected with control or TAZ siRNA. After 48 h, cell lysates were immunoblotted with the indicated antibodies. (**E**) MCF7 cells were treated with 10 μM simvastatin and the expression of each gene was assessed by RT-qPCR. The expression levels of *survivin* were normalized using the expression level of *β-actin* mRNA. Data represent the mean ± S.D. (*n* = 3). (**F**) MCF7 cells were treated with 10 μM simvastatin for 24 h. Cell lysates were then analyzed by Western blotting with the indicated antibodies. Significant differences are indicated as ** *p* < 0.01. n.s., not significant.

To investigate whether statins affect TAZ expression, the human breast cancer cell line MCF7 was treated with simvastatin (Figure 1A) or fluvastatin (Figure 1B) for 6 and 24 h. TAZ protein levels were assessed through immunoblotting. TAZ protein levels decreased after 6 h of treatment with simvastatin, and this effect persisted for 24 h (Figure 2A). Similar results were obtained for fluvastatin (Figure 2B). On the other hand, simvastatin did not markedly affect TAZ mRNA expression, although a slight increase was observed after 24 h of treatment (Figure 2C). Similar results were obtained for the human osteosarcoma cell line U2OS (Figure 2A,C). Survivin is a target gene of TAZ [4], and its knockdown reduced the expression of survivin in the present study (Figure 2D). Simvastatin decreased the protein and mRNA levels of survivin (Figure 2E,F). These results suggest that statins reduced the expression of TAZ at the protein level and suppressed its transcriptional activity.

### 2.2. Simvastatin Enhances p53 Target Gene Expression

**Figure 3:**
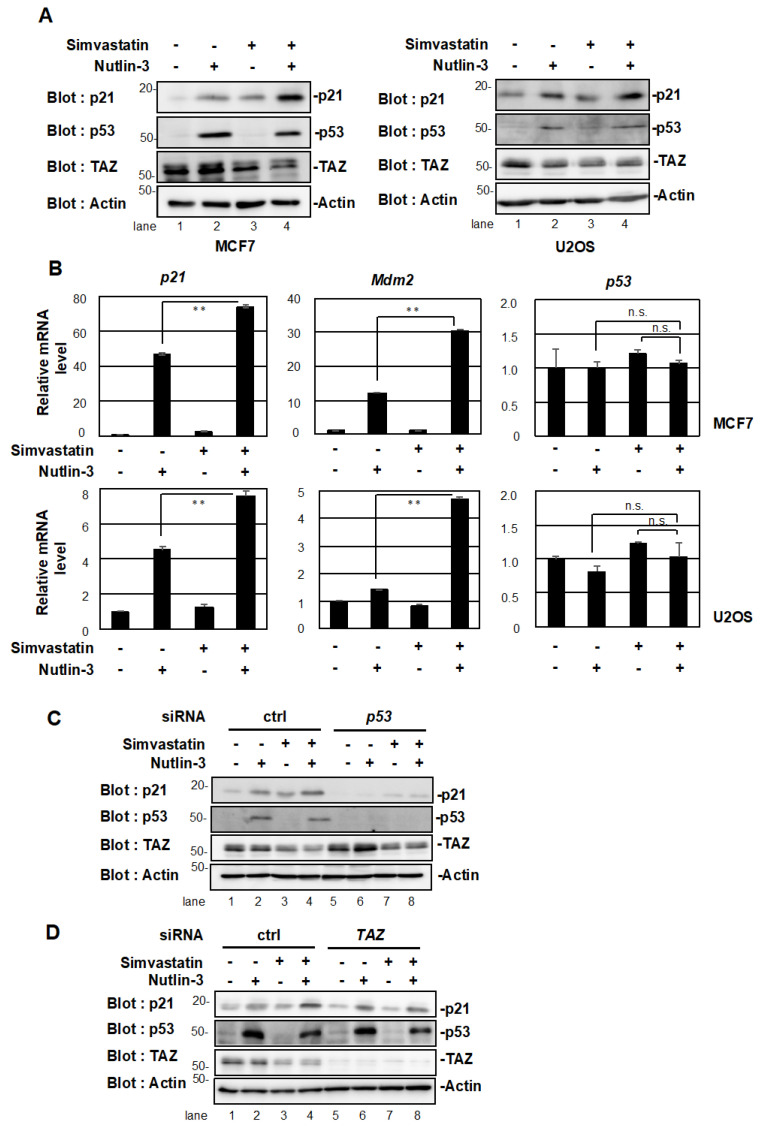
Simvastatin enhanced p53 target gene expression. (**A**) MCF7 and U2OS cells were pretreated with 10 μM simvastatin for 24 h. Cells were then incubated with 10 μM nutlin-3 for 4 h. Cell lysates were analyzed by Western blotting with the indicated antibodies. (**B**) MCF7 and U2OS cells were pretreated with 10 μM simvastatin for 24 h. Cells were then incubated with 10 μM nutlin-3 for 4 h. The expression of each gene was assessed using qPCR. The expression levels of each mRNA were normalized using the expression level of *β-actin* mRNA. Data represent the mean ± S.D. (*n* = 3). Significant differences are indicated as ** *p* < 0.01, n.s., not significant. (**C**) MCF7 cells were transiently transfected with control or *p53* siRNA. The cells were treated with 10 μM simvastatin for 24 h and then incubated with 10 μM nutlin-3 for 4 h. Cell lysates were analyzed as in (**A**). (**D**) MCF7 cells were transiently transfected with control or *TAZ* siRNA. The cells were treated with 10 μM simvastatin for 24 h and were then incubated with 10 μM nutlin-3 for 4 h. Cell lysates were analyzed as in (**A**).

We previously reported that TAZ suppressed the transcriptional activation ability of p53 [15]. Since simvastatin suppressed TAZ protein expression, we examined whether simvastatin affected the transcriptional activity of p53. In the absence of stress, p53 binds to the ubiquitin ligase Mdm2 and undergoes degradation. Nutlin-3 (Figure 1C), an inhibitor of Mdm2, activates p53 by dissociating it from Mdm2 [25]. We treated MCF7 and U2OS cells expressing WT p53 with nutlin-3 or simvastatin. As shown in Figure 3A, the protein expression of p21, a target gene of p53, was increased by the stimulation with simvastatin (lane 3) or nutlin-3 (lane 2). Furthermore, the co-treatment with nutlin-3 and simvastatin increased the expression of p21 significantly more than the single treatment (lane 4). The co-treatment with nutlin-3 and fluvastatin also increased the expression of p21 significantly more than the single treatment (Figure S1A). Similar results were observed when we examined the mRNA levels of p21 and Mdm2, the target genes of p53 (Figure 3B). On the other hand, simvastatin and nutlin-3 did not affect p53 mRNA levels.

Since simvastatin induced the expression of p21 and Mdm2, we investigated whether this effect was dependent on p53. The knockdown of p53 suppressed the up-regulation of p21 expression by the single treatment with Nutlin-3 or simvastatin and also that by the co-treatment (Figure 3C, lane 5-8). The same results were obtained for fluvastatin (Figure S1B). In addition, the knockdown of TAZ increased the induction of p21 by nutlin-3 (lane 6 versus lane 2) and slightly suppressed its induction by simvastatin (lane 7 versus lane 3). Furthermore, the up-regulation of p21 expression by the simvastatin/nutlin-3 co-treatment was suppressed to the level of nutlin-3 alone (Figure 3D, lane 8). These results suggest that statins induced the expression of p21 in a p53/TAZ-dependent manner and activated p53 by suppressing TAZ.

### 2.3. Simvastatin Enhances the Transcriptional Activation of p53

**Figure 4:**
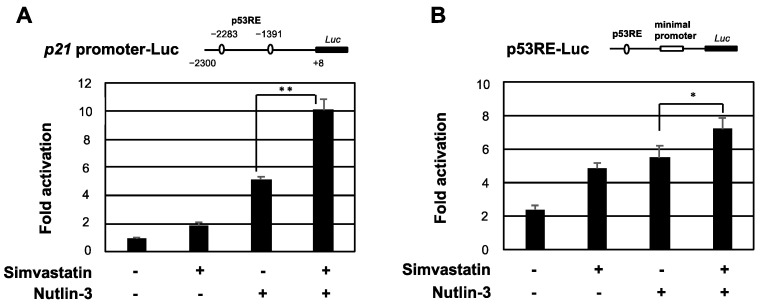
Simvastatin enhances the transcriptional activation of p53. (**A**,**B**) MCF7 cells were transfected with a construct combining the indicated reporter plasmid and pCMV/β-gal. After 24 h, cells were treated with 10 μM nutlin-3 in the presence or absence of 3 μM simvastatin for 6 h. Luciferase activity in cell lysates was measured and normalized by β-gal activity. Experiments were performed in triplicate and data were expressed as the mean activation factor ± S.D. Significant differences are indicated as ** *p* < 0.01, * *p* < 0.05.

Since simvastatin induced p21 in a p53-dependent manner, we investigated whether simvastatin enhanced the transcriptional activation ability of p53. MCF7 cells were transfected with a luciferase reporter plasmid containing the p53-binding sites of the *p21* promoter and were then subjected to a reporter assay. As expected, the promoter activity of *p21* was enhanced by nutlin-3 or simvastatin and was further promoted by the co-treatment (Figure 4A). Similar results were obtained for p53RE-Luc, which contains the p53 response sequence (p53RE) (Figure 4B). These results indicate that simvastatin induced the expression of p53 target genes by increasing the transcriptional activation ability of p53.

### 2.4. The Co-Treatment with Simvastatin and Nutlin-3 Efficiently Reduces Cancer Cell Survival

**Figure 5:**
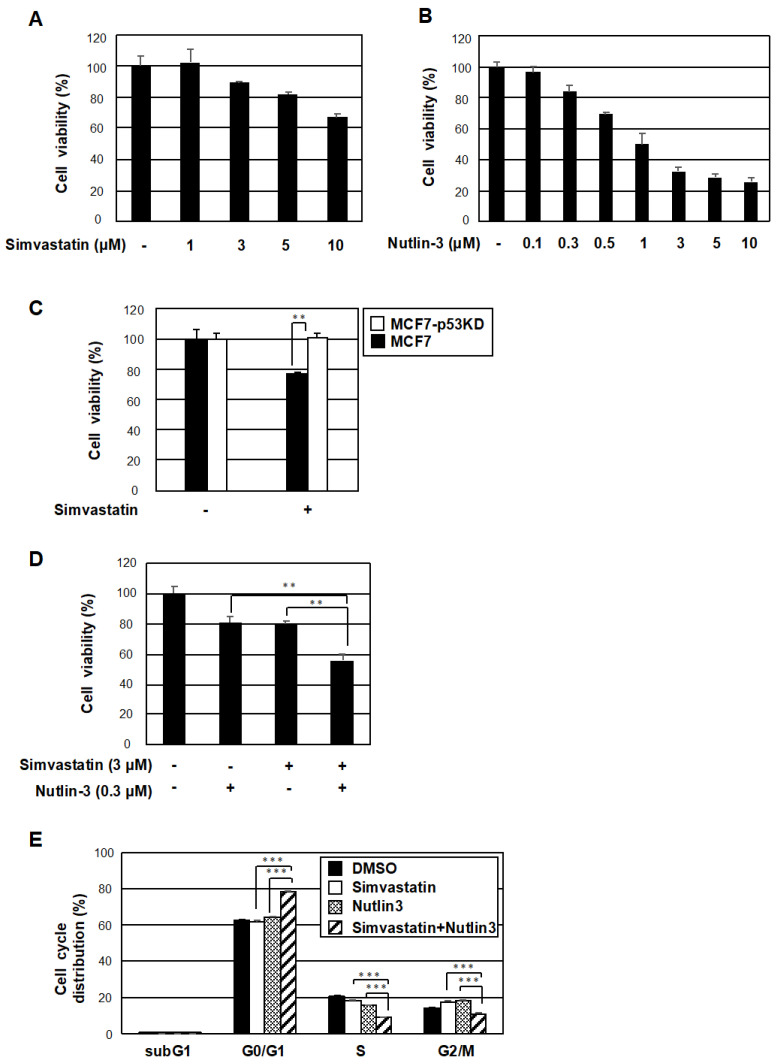
The co-treatment with simvastatin and nutlin-3 efficiently reduced cancer cell survival. (**A**) MCF7 cells were treated with the indicated concentrations of simvastatin. After 48 h, cell viability was measured with a WST-8 cell proliferation assay. Results are shown as means ± S.D. (*n* = 3). (**B**) MCF7 cells were treated with the indicated concentrations of nutlin-3. Cells were then analyzed as in (**A**). Results are shown as means ± S.D. (*n* = 3). (**C**) MCF7 wild-type cells or MCF7 cells that express shRNA to *p53* (p53KD) were treated with the 10 μM simvastatin. Cells were then analyzed as in (**A**). Results were shown as means ± S.D. (*n* = 3). (**D**) MCF7 cells were treated with 0.3 μM nulin-3 in the presence or absence of 3 μM simvastatin. Cells were then analyzed as in (A). Results were shown as means ± S.D. (*n* = 3). Significant differences are indicated as ** *p* < 0.01. (**E**) MCF7 cells were treated with 0.5 μM nulin-3 in the presence or absence of 7 μM simvastatin. After 48 h, cells were stained with propidium iodide for a flow cytometric analysis of DNA content. Significant differences are indicated as ** *p* < 0.01, *** *p* < 0.001.

We investigated whether the activation of p53 by simvastatin through the repression of TAZ expression affected the survival of cancer cells using MCF7 cells. A single treatment with nutlin-3 or simvastatin for 48 h resulted in a concentration-dependent decrease in cell viability (Figure 5A,B). However, simvastatin failed to inhibit cell proliferation in p53-knockdown MCF7 cells (Figure 5C). Importantly, the co-treatment with simvastatin and nutlin-3 reduced cell viability more efficiently than the single treatment (Figure 5D). Therefore, when changes in the cell cycle were examined, the co-treatment with simvastatin and nutlin-3 increased the G0/G1 fraction and decreased the S and G2/M fractions in MCF7 cells (Figure 5E). These results suggest that simvastatin potentiated G0/G1 arrest by nutlin-3. The combination of these two drugs may be useful in cancer therapy because they inhibited cancer cell growth in a coordinated manner.

## 3. Discussion

**Figure 6:**
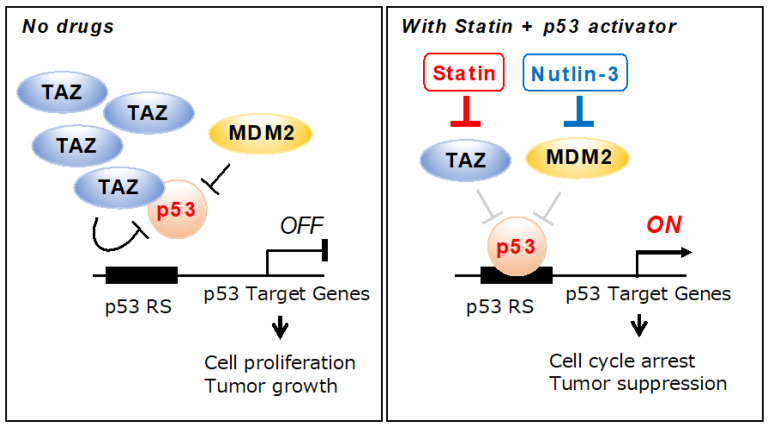
Schematic representation of a mechanistic model for TAZ-mediated activation of p53 by statin. The statin facilitated p53 transcriptional activity by repressing TAZ expression. Statins that decrease the p53-TAZ interaction in tumor cells that overexpress TAZ proteins may increase the cellular sensitivity to chemotherapeutic agents, such as p53 activators, inducing a stronger p53 response and suppressing tumor cell survival.

Statins are used to treat hyper-LDL-cholesterolemia through the inhibition of HMG-CoA reductase [16]. Since the effects of simvastatin and fluvastatin are mild, they are the most commonly used statins in clinical practice. Both statins are highly liposoluble and easily transferred to various tissues. Therefore, they are expected to exert additional effects besides lowering cholesterol. The repositioning of simvastatin as an anti-tumor agent has recently attracted attention because of a reduction in the mortality rate of cancer patients taking simvastatin [16,26]. Simvastatin has been reported to inhibit the growth and survival of cancer cells; however, the mechanisms by which it exerts its anti-tumor effects have not yet been elucidated. In the present study, we demonstrated that simvastatin and fluvastatin activated p53, a tumor suppressor gene, by suppressing TAZ protein expression. Other statins, such as mevastatin and lovastatin, also strongly inhibit TAZ and are expected to exert similar effects [22]. The result showing that statins activate p53 by suppressing TAZ protein expression and inhibiting cancer cell growth provides insights into the mechanisms underlying the antitumor effects of statins (Figure 6).

In MCF7 cells, the co-treatment with simvastatin and nutlin-3 cooperatively up-regulated the expression of p21, a target gene of p53. The knockdown of p53 abolished the cooperative effect, suggesting that simvastatin regulates the activity of p53. However, simvastatin did not regulate the expression of p53 at the mRNA or protein level, indicating that simvastatin regulates the functions, but not the amount, of p53. The functions of p53 are tightly regulated by post-translational modifications, such as acetylation, phosphorylation, and methylation [9,27]. p53 acetylation directly affects the transcriptional activity of p53 by altering the structure of the protein and regulating its binding to gene targets [28,29,30]. The methylation of p53 is also required for nuclear localization and binding to DNA [31,32]. Further studies are needed to investigate the post-translational modifications induced in p53 by simvastatin in order to validate its effects.

We speculate that simvastatin indirectly rather than directly activates p53. The knockdown of TAZ suppressed the cooperative up-regulation of p21 by simvastatin and nutlin-3 to the same extent as nutlin-3 alone. These results suggest that simvastatin potentiates p53 activators more efficiently by suppressing the activity of TAZ. In a previous study, we found that TAZ suppressed p53 functions [15]. Simvastatin and fluvastatin reduced TAZ protein levels (Figure 2A,B), suggesting that the destabilization of the TAZ protein by statins enhanced the transcriptional activation of p53. We speculate that the destabilization of TAZ is due to simvastatin repressing the activities of Ras and Rho and LATS1/2 promoting the phosphorylation of TAZ. The phosphorylation of TAZ localizes it to the cytoplasm, where it is degraded by ubiquitin ligases, such as SCF^β-TrCP^ [33,34].

Simvastatin suppressed cancer cell survival in a concentration-dependent manner. However, simvastatin did not affect the survival of MCF7 cells with the knockdown of p53. Statins have been suggested to contribute to cancer cell survival through p53 functions. Neither nutlin-3 nor simvastatin affected cell growth at low concentrations. However, even at low concentrations, the co-treatment markedly reduced cell proliferation. Nutlin-3 arrests the G1 phase by increasing p21 expression levels via p53, causing an increase in the G1 phase fraction and a decrease in the S phase fraction [35]. Importantly, the combination with statins enhanced the effects of G1 arrest through the activation of p53 and suppressed cancer cell survival. If statins contribute as a p53 activator, side effects may be attenuated and therapeutic effects may be enhanced by reducing the dosages of drugs, such as nutlin-3 and actinomycin D.

## 4. Materials and Methods

### 4.1. Materials

Simavastatin (10010344) and (-)-nutlin-3 (18585) were purchased from Cayman Chemicals (Ann Arbor, MI, USA). Fluvastatin (068-06641) was purchased from Fujifilm Wako (Osaka, Japan).

### 4.2. Cell Lines, Plasmids, and Transfection

MCF7 (WT p53) cells and U2OS (WT p53) cells were cultured in High-Glucose Dulbecco’s Modified Eagle’s Medium (Sigma, St. Louis, MO, USA) supplemented with 10% heat-inactivated fetal bovine serum (FBS) (Sigma), 100 U/mL of penicillin G, and 100 μg/mL of streptomycin at 37 °C in the presence of 5% CO_2_ [36]. H1299 (p53-null) cells were cultured in Roswell Park Memorial Institute 1640 medium (Sigma) supplemented with 10% heat-inactivated FBS and penicillin/streptomycin [15]. The MCF7-shp53 cells were previously established have been described [37].

The original constructs encoding p53RE-Luc (pGL4/p53RE) and *p21* promoter-Luc (pGL4/p21) have been previously described [38,39]. Human *TAZ* siRNA (sense: 5′-AGACAUGAUCCAUCACUAA-3′) was purchased from FASMAC (Kanagawa, Japan). The siRNA oligo targeting human *p53* mRNA has been previously described [15]. Stealth RNAi™ siRNA Negative Control Med GC Duplex was obtained from Invitrogen (Waltham, MA, USA).

### 4.3. RNA Extraction, Reverse Transcription, and Quantitative PCR (qPCR)

Total RNA was extracted as previously described [15]. cDNA was synthesized with ReverTra qPCR RT Master Mix (TOYOBO, Osaka, Japan) according to the manufacturer’s instructions. qPCR was performed with TB Green Premix Ex Taq II (TaKaRa Bio Inc., Shiga, Japan) using the ABI Prism 7300 sequence detection system (Applied Biosystems, South San Francisco, CA, USA) [15]. A dissociation curve consisting of a single peak was used to confirm the specificities of the detected signal. *β-actin* was used as the internal control. The following primer sequences were used: human *p21*: forward, 5′-GATTTCTACCACTCCAAACGCC-3′, reverse, 5′-AGAAGATGTAGAGCGGGC-3′; human *Mdm2*: forward, 5′-TGTTGGTGCACAAAAAGACA-3′, reverse, 5′-CACGCCAAACAAATCTCCTA-3′; human *Survivin*: forward, 5′-AGAACTGGCCCTTCTTGGAGG-3′, reverse, 5′-CTTTTTATGTTCCTCTATGGGGTC-3′; human *p53*: forward, 5′-TTTGCTTTCCTTGGTCAGGC-3′, reverse, 5′-GCTTGCGACCTTGACCAT CT-3′; human *TAZ*: forward, 5′-GGCTGGGAGATGACCTTCAC-3′, reverse, 5′-CTGAGTGGGGTGGTTCTGCT-3′.

### 4.4. Immunochemical Methods and Antibodies

Protein solubilization and immunoblotting were conducted as previously described [15]. The following commercially available antibodies were used: anti-TAZ (V386) (#4883; Cell Signaling Technology, Beverly, MA, USA), anti-survivin (71G4B7) (#2808; Cell Signaling Technology), anti-p53 (sc-126; Santa Cruz Biotechnology, Santa Cruz, CA, USA), anti-p21 (F-5, Santa Cruz Biotechnology), and anti-β-actin-HRP (C4; Santa Cruz Biotechnology).

### 4.5. Reporter Assay

The luciferase reporter plasmid, expression plasmids, pCMV/β-gal, and an empty vector were transfected into MCF7 cells. In each experiment, the total amount of DNA transfected was the same. After 24 h, cells were treated with the indicated drugs for 6 h and luciferase activity was measured. Luciferase activity was normalized using β-gal activity [40].

### 4.6. Cell Viability Assay

Cell viability was evaluated using a Cell Counting Kit-8 (Dojindo, Kumamoto, Japan) [41]. At a concentration of 5 × 10^3^ cells/well, cells were seeded into 96-well plates. After 24 h, cells were treated with simvastatin and/or nutlin-3. After 48 h, the cells were incubated at 37 °C for 3 h in a humidified atmosphere of 5% CO_2_ in the presence of WST-8. Absorbance at 450 nm by the medium was measured using a microplate reader (Multiskan FC, Thermo Fisher Scientific, Waltham, MA, USA).

### 4.7. Cell Cycle Analysis

To observe the cell cycle phase distribution, cells were stained with propidium iodide using the Cycletest^TM^ Plus DNA Kit (BD Biosciences, Franklin Lakes, NJ, USA). Changes in intracellular DNA content were detected using the FACSVerse^TM^ flow cytometer (BD Biosciences). The analysis was performed with FACSuite^TM^ software (BD Biosciences).

### 4.8. Statistical Analysis

A two-tailed Student’s *t*-test was used to evaluate the significance of differences between two groups. A one-way analysis of variance with the post hoc Tukey–Kramer honest significant difference test was used to assess significance in multigroup analyses.

## 5. Conclusions

In the present study, we show that simvastatin and fluvastatin activated p53 by suppressing TAZ functions. The combination of statins such as simvastatin with antitumor agents that target p53 is effective in cancers in which the *TP53* gene is normal but its function is suppressed by high expression of TAZ. 

## Data Availability

Data are contained within the article and Supplementary Materials.

## Supplementary Materials

The following supporting information can be downloaded at: https://www.mdpi.com/article/10.3390/ph15081015/s1, Figure S1: Fluvastatin induces p21 expression via p53.
