# Supplementary figures and images for: HMG-CoA Reductase Inhibitor Statins Activate the Transcriptional Activity of p53 by Regulating the Expression of TAZ
Source: Pharmaceuticals (Basel). 2022 Aug 17;15(8):1015. doi: 10.3390/ph15081015 (PMC9412369; doi:10.3390/ph15081015)

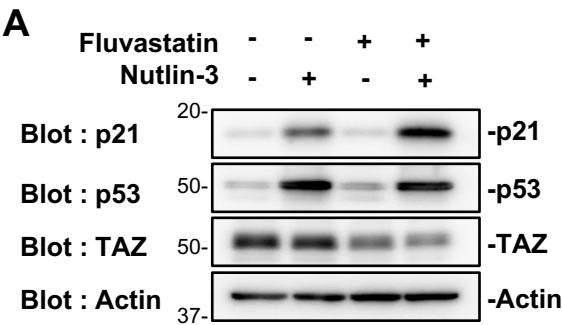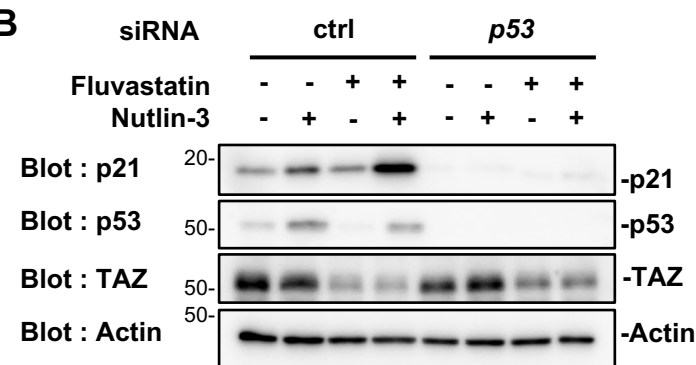

Figure S1

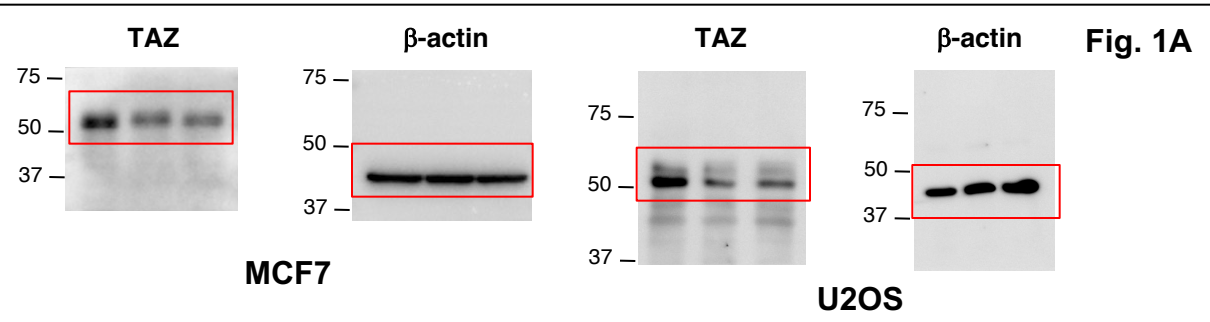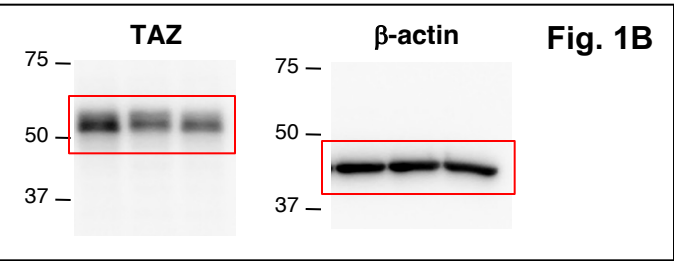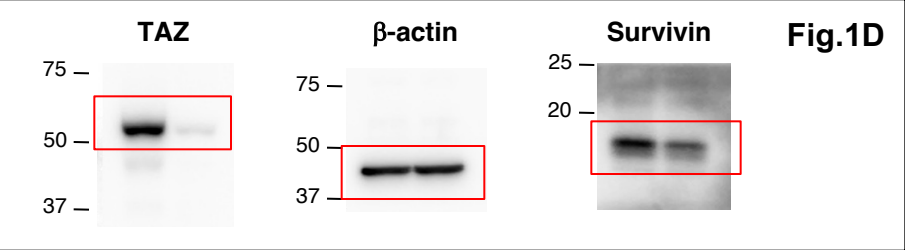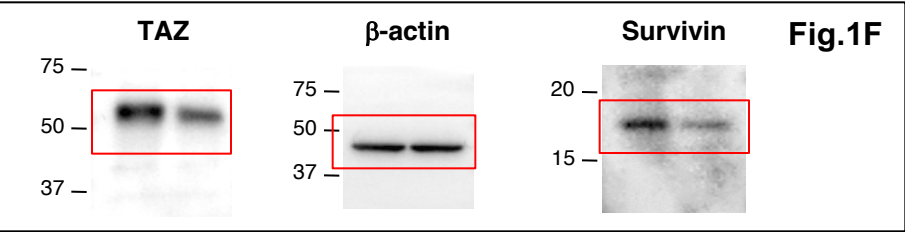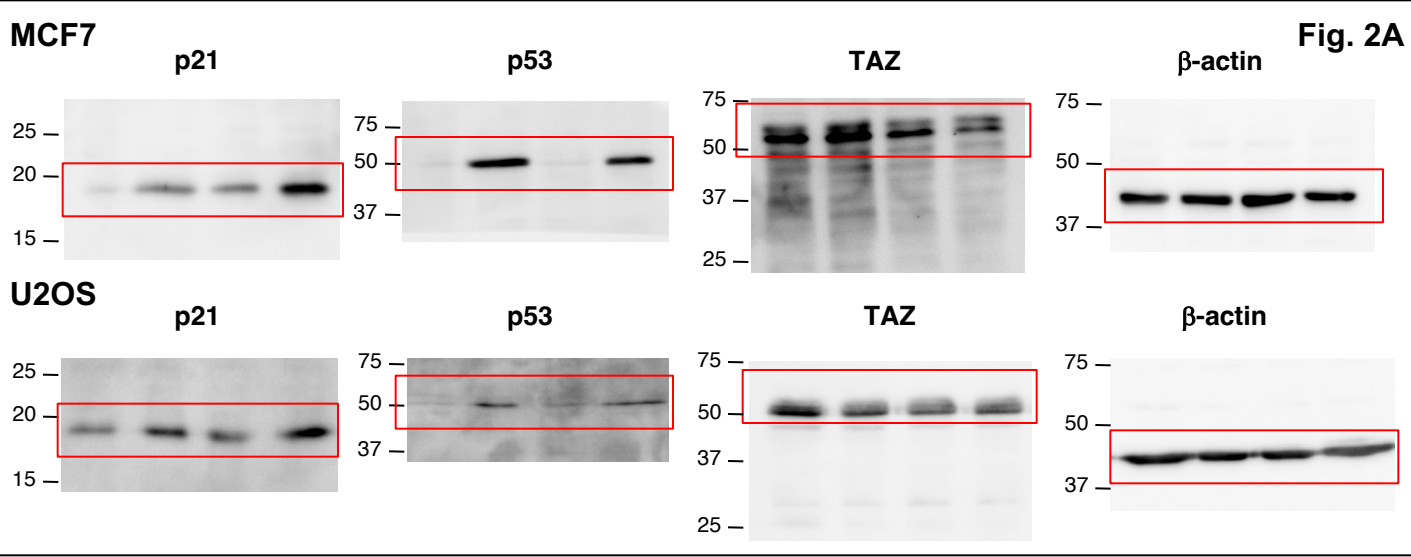

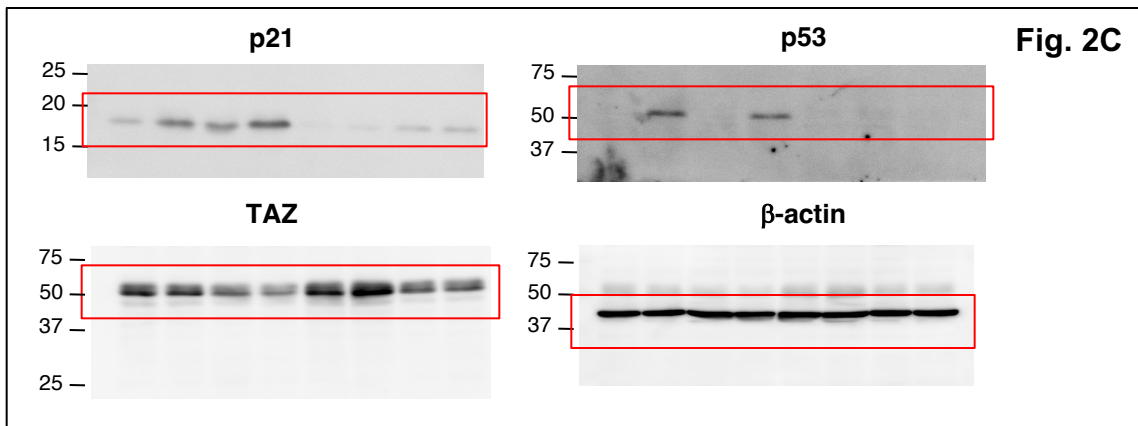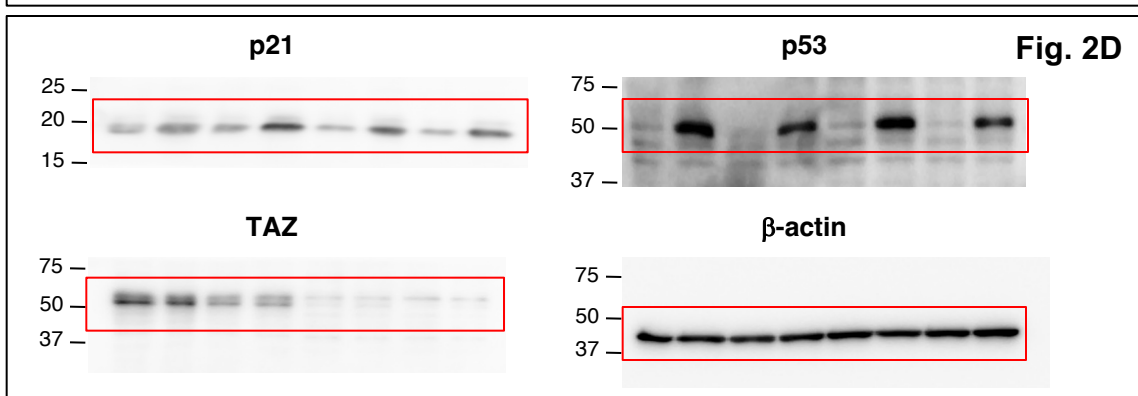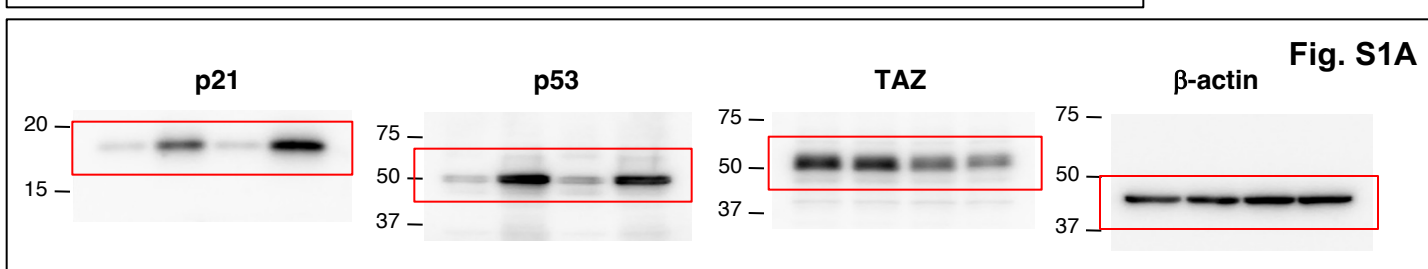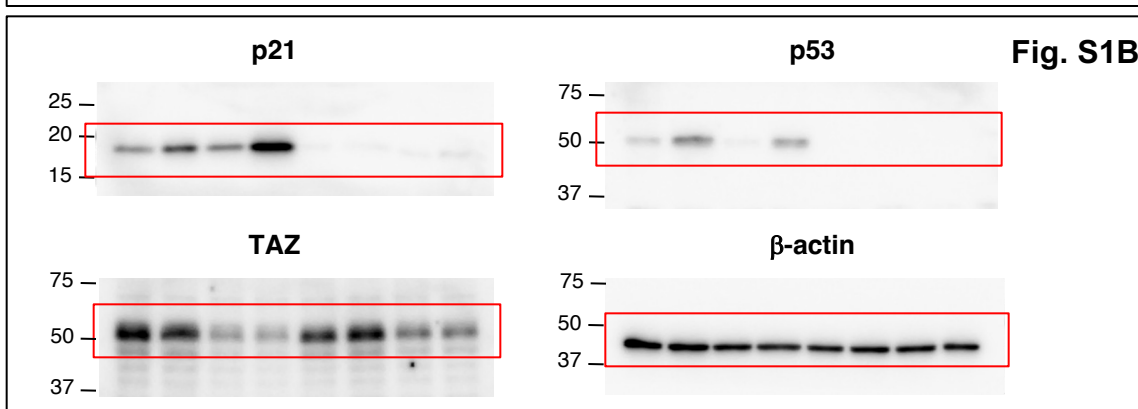

Supplement: Supplementary file 1 [file pharmaceuticals-15-01015-s001.zip › pharmaceuticals-1847429-supplementary.pdf]
